# Bisdemethoxycurcumin attenuates gastric adenocarcinoma growth by inducing mitochondrial dysfunction

**DOI:** 10.3892/ol.2014.2685

**Published:** 2014-11-07

**Authors:** CHANGJIANG LUO, ZHIXING DU, XING WEI, GANG CHEN, ZHONGXUE FU

**Affiliations:** 1 Department of Gastrointestinal Surgery, The First Affiliated Hospital, Chongqing Medical University, Chongqing 400016, P.R. China; 2 Department of General Surgery, Lanzhou University Second Hospital, Lanzhou, Gansu 730030, P.R. China; 3 Department of Surgical ICU, Lanzhou University Second Hospital, Lanzhou, Gansu 730030, P.R. China

**Keywords:** bisdemethoxycurcumin, gastric adenocarcinoma, tumor growth inhibition, apoptosis, mitochondria

## Abstract

Bisdemethoxycurcumin (BDMC) is a demethoxy derivative of curcumin. In this study, a human gastric adenocarcinoma xenograft model was generated *in vivo* using nude mice and BDMC was observed to suppress the growth and activity of tumors, in addition to improving the physical and mental capacity of the mice. An increased number of apoptotic cells, decreased ratio of B-cell lymphoma 2 (Bcl-2)/Bcl-2-associated X protein and increased caspase-3 expression was also observed following treatment with BDMC, indicating that BDMC may promote apoptosis in tumors via mitochondrial modulation. The growth of SGC 7901 gastric cancer cells was inhibited and arrested at G_1_ phase. Specific indicators of mitochondrial dysfunction, a reduction in adenosine triphosphate generation, the inner mitochondrial membrane potential, augmentation of reactive oxygen species production and cytochrome c were also detected in the mitochondria following treatment with BDMC. These results indicate that BDMC attenuates gastric adenocarcinoma growth by inducing mitochondrial dysfunction.

## Introduction

Gastric carcinoma is one of the most common types of cancer worldwide, which may arise from any section of the stomach. It accounts for ~800,000 mortalities worldwide annually, with a poor prognosis (five-year survival rate, <5–15%). Adenocarcinoma of the stomach is the most frequent type of gastric carcinoma, representing 90–95% of cases. Certain drugs, including, fluorouracil, cytarabine and tegafur, have been effective in the inhibition of carcinoma growth. However, the toxicity of these drugs is high and certain patients do not tolerate the side effects well (1). Therefore, the identification of novel drug molecules with low toxicity for the treatment of gastric adenocarcinoma is required. Previous studies have focused on the anticancer effects of natural products, which are considered to exhibit reduced toxicity.

Bisdemethoxycurcumin (BDMC) is a demethoxy derivative of curcumin, which is a natural substance found in the turmeric root. The other two curcuminoids are demethoxycurcumin and curcumin, which have been reported to suppress the proliferation of numerous cancer cells (2). BDMC is significantly more stable than curcumin in physiological media. However, whether it arrests the cell cycle of gastric adenocarcinoma and attenuates gastric adenocarcinoma growth remains unclear. The current study investigated the effect of BDMC on the growth inhibition of gastric adenocarcinoma *in vivo* and *in vitro*. As mitochondria are critical for the growth of tumors, the mitochondrial function of gastric adenocarcinoma cells following BDMC treatment was analyzed.

## Materials and methods

### Tumor xenograft generation and size detection

Human gastric adenocarcinoma tissue was obtained from a 65 year old male patient (T2N1M0, stage IB) during radical surgery at Lanzhou University Second Hospital (Lanzhou, China). Written informed consent was obtained from the patient. The tissue was cut into 2.5×2.5×2.5 mm sections, rinsed with RPMI-1640 medium (Gibco-BRL, Carlsbad, CA, USA) and subcutaneously injected into the right lower limbs of 24 male nude mice (age, 2 months old; weight, 20±2 g; Animal Research Center, Lanzhou University, Lanzhou, China). The mice (tumor size, >150 mm^3^) were used 12 days following transplantation. In total, 12 mice were symmetrically subcutaneously injected around the xenograft with 100 mg/kg/day BDMC dissolved in dimethyl sulfoxide (DMSO; Sigma-B6938, Sigma-Aldrich, St. Louis, MO, USA) for three weeks. The tumor sizes were measured every three days for 21 days and were calculated using the following formula: Tumor size (v, mm^3^) = π/6 × ax b^2^ (a, major axis, mm; b, minor axis, mm^2^) (3). This study was approved by the ethics committee of Lanzhou University Second Hospital (Lanzhou, China).

### Pathology and apoptosis detection

Gastric adenocarcinomas transplanted into two groups of nude mice were collected after 21 days and cut into 6 μm sections transversely. Pathological analysis was conducted using hematoxylin and eosin staining and apoptotic analysis was performed using terminal deoxynucleotidyl transferase dUTP nick end labeling (TUNEL) (QIA33 FragEL™ DNA Fragmentation Detection Kit, Merck KGaA, Darmstadt, Germany) following the manufacturer’s instructions. Immunohistochemistry (IHC) was performed to detect apoptotic related factors. The primary antibodies used were anti-human anti-B-cell lymphoma 2 (Bcl-2 )[B7025; Anbobio Biotechnology, Co., Ltd., San Francisco, CA, USA; rabbit immunoglobulin polyclonal (Ig)G, 1:100], anti-Bcl-2-associated X protein (Bax) (C0132; Anbobio Biotechnology, Co., Ltd.; rabbit polyclonal IgG, 1:100), anti-caspase-3 (ab2302; Abcam, Cambridge, UK; Rabbit polyclonal IgG, 1:200) while secondary antibody was goat anti-rabbit polyclonal IgG (AP307P; horseradish peroxidase-conjugate; Millipore, Billerica, MA, USA; 1:10,000). Images were captured using a confocal microscope (Nikon TE2000; Nikon Corporation, Tokyo, Japan).

### Cytotoxicity and cell cycle analysis

Gastric cancer cells (cell line SGC 7901) were seeded into a 96-well cell culture plate (Corning Inc., New York, NY, USA) at a density of 3,000 cells/well and treated with final concentration of 1, 5, 20, 50, 100 or 200 μM BDMC dissolved in DMSO, for 72 h. Cytotoxicity was measured using an XTT cell viability assay (Sigma-Aldrich) according to the manufacturer’s instructions. Cell cycle analysis of SGC 7901 cells treated with 100 μM BDMC was performed using flow cytometry (FCM; BD FACSArray; BD Biosciences, Franklin Lakes, NJ, USA).

### Effect of BDMC on mitochondrial function

Cells were seeded into six-well plates (Corning, Inc.) and treated with 100 μM BDMC for 24 h. Cells were then washed with phosphate-buffered saline (PBS), and incubated in PBS containing 10 μM fluorescent probe, dichlorodihydrofluorescein diacetate (Sigma-Aldrich) for 30 min at 37°C prior to determination of reactive oxygen species (ROS) levels and then analyzed by FCM. JC-1 was used to determine the inner mitochondrial membrane potential (Δψm). Cells were incubated in RPMI-1640 (Gibco-BRL) containing cationic carbocyanine dye, JC-1 (Sigma-Aldrich; 5 μg/ml), for 30 min at 37°C then analyzed by FCM. The Δψm was calculated as the red/green fluorescent ratio. The adenosine triphosphate (ATP) concentration in the mitochondrial fraction and the cytochrome *c* (Cyt *c*) levels in the mitochondrial and cytosolic fractions of the cells were measured using reverse-phase high-performance liquid chromatography (HPLC 1100; Agilent Technologies, Palo Alto, CA, USA). The translocation of Cyt *c* was assessed using its ratio in the mitochondrial and cytosolic fractions following the manufacturer’s instructions.

### Data analysis

Each experiment was performed at least in triplicate. Data was analyzed using SPSS version 12.0 (SPSS, Inc., Chicago, IL, USA) and presented as the mean ± standard deviation. The Student’s *t* test was used to evaluate two groups and one-way analysis of variance was used for multi-groups. P<0.05 was considered to indicate a statistically significant difference.

## Results

### Analysis of tumor size and pathology

**Figure 1:**
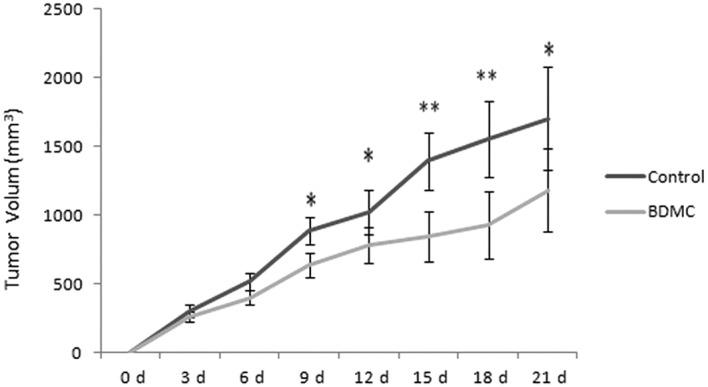
Tumor volumes of xenograft model on nude mice in control and BDMC groups. ^*^P<0.05 and ^**^P<0.01. BDMC, bisdemethoxycurcumin.

**Figure 2:**
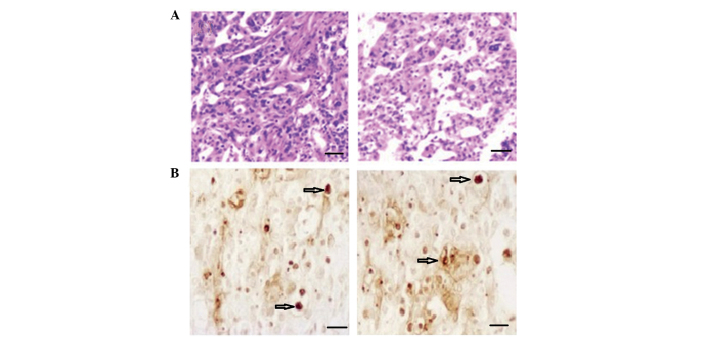
(A) Pathological analysis of tumors of a xenograft model in nude mice in control (left) and BDMC (right) groups. (hematoxylin and eosin staining; magnification, ×100). The bar is equivalent to 100 μm. (B) Apoptotic cells were visualized using terminal deoxynucleotidyl transferase dUTP nick end labeling in control (left) and BDMC (right) groups. (magnification, ×200). The bar is equalivent to 50 μm. BDMC, bisdemethoxycurcumin.

The xenograft model was generated successfully in nude mice and the tumors grew rapidly without treatment. However, the tumor size was decreased in the BDMC group when compared with the control group (Fig. 1). Additionally, during animal feeding, on observation the physical and mental capacity of the mice in the BDMC group appeared to improve. Pathological analysis indicated increased hyperplasia in the tumors with high density cell nuclei and abundant cytoplasm in the control group. Low density infiltration and smaller cell nuclei were identified in the BDMC group, in addition to low activity in the tumor tissues (Fig. 2A). BDMC suppressed the growth and activity of the tumor cells.

### Apoptotic and relative factor analysis of the tumor

**Figure 3:**
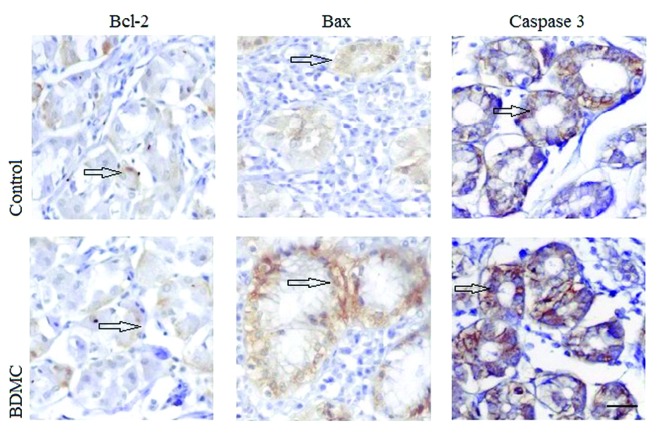
Analysis of the expression of apoptotic related factors Bcl-2, Bax and caspase-3 in control and BDMC groups. (magnification, ×400). The bar is equivalent to 25 μm. Bcl-2, B-cell lymphoma 2; Bax, Bcl-2 associated X protein; BDMC, bisdemethoxycurcumin.

**Figure 4:**
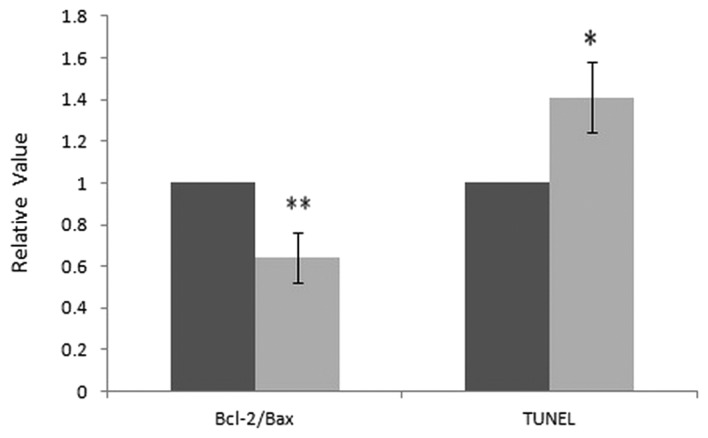
Relative ratios of Bcl-2/Bax and TUNEL positive cells in control and BDMC groups. Left: ratio of Bcl-2/Bax; right: apoptosis cells (TUNEL positive cells).^*^P<0.05 and ^**^P<0.01. TUNEL, terminal deoxynucleotidyl transferase dUTP nick end labeling; Bcl-2, B-cell lymphoma 2; Bax, Bcl-2 associated X protein; BDMC, bisdemethoxycurcumin.

An increased number of apoptotic cells (Fig 2; arrow indicated) were detected using TUNEL in the BDMC group. The results indicated that tumor apoptosis was enhanced following treatment with BDMC. Images are shown in Fig. 2B. IHC was used to detect the apoptotic factors *in situ*. As shown in Fig. 3, cytoplasmic Bcl-2 levels were decreased, while cytoplasmic Bax and nuclear caspase-3 levels were increased in the BDMC group when compared with that of the control group. The relative values of TUNEL positive cells and the relative ratio of Bcl-2/Bax was analyzed, where the control group was set as 1, and the relative values in the BDMC group were compared with the control, as shown in Fig. 4. These result indicate that BDMC may promote apoptosis in gastric adenocarcinoma via mitochondria mediated Bcl-2 family and caspase pathways.

### Cell viability and cell cycle analysis

**Figure 5:**
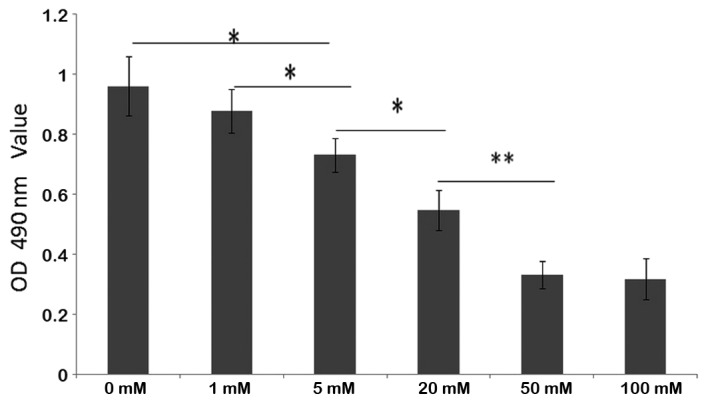
Cell viability detections in control and BDMC groups. ^*^P<0.05 and ^**^P<0.01. BDMC, bisdemethoxycurcumin; OD, optical density.

BDMC exhibited dose-dependent cytotoxic effects on the growth of SGC 7901 cells. The viability of SGC 7901 cells treated with BDMC at concentrations of >5 μM was significantly inhibited. No evident increase in inhibition was identified in the groups treated with >100 μM (Fig. 5). A dose of 100 μM BDMC was determined to be the most efficacious concentration. Following treatment with 100 μM BDMC, the percentage of cells at G_1_ phase in the BDMC group (62.9±5.5%) was increased significantly when compared with that of the control group (53.4±6.0%) (P<0.05). The results indicated that BDMC may inhibit cell growth and arrest the cell cycle at G_1_ phase.

### Effects of BDMC on mitochondrial function

**Figure 6:**
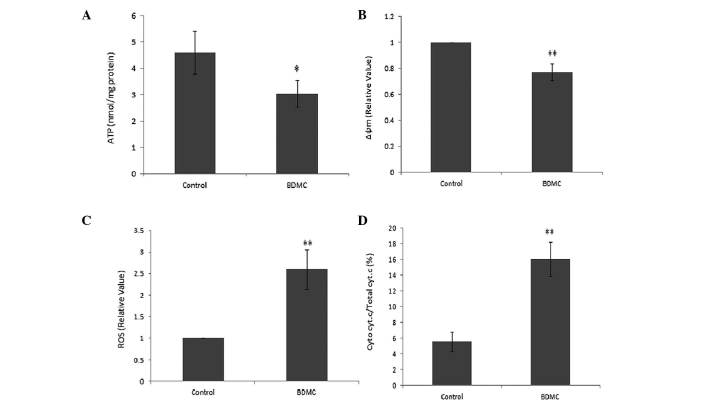
Detection of mitochondrial function in control and BDMC groups. (A) The generation of ATP was markedly decreased in SGC 7901 cells following treatment with BDMC. (B) BDMC caused a reduction in Δψm. (C) increases in ROS generation in SGC 7901 cells were identified following treatment with BDMC. (D) An increased ratio of Cyt *c* in the mitochondrial and cytosolic fractions was observed. ^*^P<0.05 and ^**^P<0.01. BDMC, bisdemethoxycurcumin.

The generation of ATP was markedly decreased in SGC 7901 cells following treatment with BDMC (Fig. 6A). The mitochondrial potential sensor, JC-1, was used to detect the Δψm, where a ~25% reduction in the ratio of red to green fluorescence was identified in the BDMC group, indicating that BDMC causes a reduction in Δψm (Fig. 6B). Rapid increases in ROS generation in SGC 7901 cells were identified following treatment with BDMC (Fig. 6C). Furthermore, the release of Cyt *c* due to mitochondrial damage caused by BMDC, was observed in the BDMC group, which resulted in an increased ratio of Cyt *c* in the mitochondrial and cytosolic fractions (Fig. 6D). These results indicated the effect of BDMC on mitochondrial dysfunction.

## Discussion

BDMC, together with curcumin and demethoxycurcumin, are the three predominant active compounds derived from the turmeric root. Previous studies have shown that curcuminoids exhibit a variety of therapeutic effects, including neointima formation (4), anti-neurodegenerative (5), anti-cancer (6) and anit-inflammatory (7) effects. However, the effects of BDMC have rarely been reported. Thus, the present study investigates the effects of BDMC on gastric carcinoma. Gastric carcinoma is the second most common type of cancer worldwide (8). It may be classified into several types, including adenocarcinoma of the stomach (90–95% of cases), which develops from the epithelial tissue of glandular origin and/or glandular characteristics, lymphoma of the stomach (4% of gastric carcinomas), gastrointestinal stromal tumor, neuroendocrine tumors, squamous cell carcinoma, leiomyosarcoma and small cell carcinoma. Treatment of adenocarcinoma of the stomach is difficult as few drugs are effective with few side effects.

In the current study, a human gastric adenocarcinoma tumor xenograft model was generated in nude mice, which were then treated with BDMC. BDMC was observed to inhibit the tumor growth and caused an increase in body weight. Pathological analysis indicated that the number of positively stained cells was reduced, indicating that BDMC reduced the rapid growth of cancer cells and decreased activity in the tumor cells. This was also observed *in vitro*. BDMC inhibits the growth of gastric cancer SGC 7901 cells in a dose-dependent manner. The most efficacious concentration for cell growth inhibition was determined as 100 mM BDMC. Cell growth is controlled by the cell cycle, which is divided into G_1_, S, G_2_ and M phase. This study revealed that BDMC arrests the cell growth in G_1_ phase and thus, cells could not reach S phase and complete the cycle. Apoptosis and inhibition of cell growth were observed in the tumor following treatment with BDMC. The apoptosis regulators, the Bcl-2 family, governs mitochondrial outer membrane permeabilization (9). The level of proapoptotic factor Bax, was increased and anti-apoptotic Bcl-2 was decreased following treatment with BDMC. Caspase-3 was also increased following treatment with BDMC. Caspase-3 is activated in apoptotic cells by mitochondrial pathways, in which Cyt *c* from the mitochondria works in combination with caspase-9, apoptosis-activating factor 1 (Apaf-1) and ATP to process procaspase-3 and subsequently activate caspase-3 (10–12). The results of this study indicated that BDMC may cause mitochondrial dysfunction in tumors.

Cancer is characterized uncontrolled cell growth. Considering that mitochondria is involved in cell proliferation and division, mitochondrial dysfunction is serious in cells and may cause cytotoxicity and cell cycle arrest (13). The mitochondrion is a membrane-bound organelle in eukaryotic cells, which generates ATP as chemical energy via respiration and the regulation of cellular metabolism. Due to their rapid growth, cancer cells require a large amount of ATP to synthesize bioactive compounds for rapid cell proliferation mainly via the oxidative phosphorylation pathway (14,15). However, following treatment with BDMC, ATP generation was decreased significantly and thus, the energy supply for rapid cell proliferation was insufficient, leading to the inhibition of cell growth. The Δψm is an important parameter of mitochondrial function and may be used as an indicator of cell growth (16). A high Δψm has been observed in rapidly proliferating cells with complex form called J-aggregate, while a low Δψm has been observed in apoptotic cells with a monomeric form of JC-1 (17). In the current study, a significant decrease in Δψm was detected following treatment with BDMC for 24 h. ROS are chemically reactive molecules, which at low levels, facilitate cancer cell survival by cell-cycle promotion; however, at higher levels may suppress tumor growth via the activation of cell-cycle inhibitors and the induction of cell death (18). Following treatment with BMDC for 4 and 24 h, the levels of ROS in the SGC 7901 gastric cancer cells increased significantly. The augmentation of ROS production may cause fatal injury to the cancer cells (19). Cyt *c* is an essential component of the electron transport chain, capable of undergoing oxidation and reduction, which transfers electrons between Complexes III (Coenzyme Q-Cyt *c* reductase) and IV (Cyt *c* oxidase), indicating its significance in cancers (20). Cyt *c* is also involved in the initiation of apoptosis, by binding to Apaf-1 (21). This study revealed increased levels of Cyt *c* and caspase-3 following treatment with BDMC, compared with the control groups. These results indicated that BMDC attenuated gastric adenocarcinoma growth by inducing mitochondrial dysfunction. Therefore, BDMC may present as a potential drug for gastric adenocarcinoma. However, further preclinical studies regarding BMDC pharmacodynamics, pharmacology and side-effects are required to identify whether BDMC may be used as an anti-cancer reatment.
